# A mutation of *EPT1* (*SELENOI*) underlies a new disorder of Kennedy pathway phospholipid biosynthesis

**DOI:** 10.1093/brain/aww318

**Published:** 2017-01-04

**Authors:** Mustafa Y Ahmed, Aisha Al-Khayat, Fathiya Al-Murshedi, Amna Al-Futaisi, Barry A Chioza, J Pedro Fernandez-Murray, Jay E Self, Claire G Salter, Gaurav V Harlalka, Lettie E Rawlins, Sana Al-Zuhaibi, Faisal Al-Azri, Fatma Al-Rashdi, Amaury Cazenave-Gassiot, Markus R Wenk, Fatema Al-Salmi, Michael A Patton, David L Silver, Emma L Baple, Christopher R McMaster, Andrew H Crosby

**Affiliations:** 1 Medical Research (Level 4), University of Exeter Medical School, RILD Wellcome Wolfson Centre, Royal Devon and Exeter NHS Foundation Trust, Barrack Road, Exeter, UK; 2 Department of Biology, College of Science, Sultan Qaboos University, Sultanate of Oman; 3 Department of Genetics, College of Medicine, Sultan Qaboos University, Sultanate of Oman; 4 Department of Paediatrics, Sultan Qaboos University Hospital, Sultanate of Oman; 5 Department of Pharmacology, Dalhousie University, Halifax, NS, Canada; 6 Faculty of Medicine, University of Southampton, UK; 7 West Midlands Regional Genetics Service, Birmingham Women’s NHS Foundation Trust, Mindelsohn Way, Birmingham, UK; 8 Department of Ophthalmology, Sultan Qaboos University Hospital, Sultanate of Oman; 9 Department of Radiology and Molecular Imaging, Sultan Qaboos University Hospital, Sultanate of Oman; 10 Department of Paediatrics, Sameal Hospital, Ministry of Health, Sultanate of Oman; 11 SLING, Life Sciences Institute, National University of Singapore, Singapore; 12 Department of Biochemistry, National University of Singapore, Singapore; 13 Signature Research Program in Cardiovascular and Metabolic Disorders, Duke–NUS Medical School, Singapore

**Keywords:** EPT1 mutation, Kennedy pathway, phospholipid biosynthesis, hereditary spastic paraplegia, whole exome sequencing

## Abstract

Mutations in genes involved in lipid metabolism have increasingly been associated with various subtypes of hereditary spastic paraplegia, a highly heterogeneous group of neurodegenerative motor neuron disorders characterized by spastic paraparesis. Here, we report an unusual autosomal recessive neurodegenerative condition, best classified as a complicated form of hereditary spastic paraplegia, associated with mutation in the ethanolaminephosphotransferase 1 (*EPT1*) gene (now known as *SELENOI*), responsible for the final step in Kennedy pathway forming phosphatidylethanolamine from CDP-ethanolamine. Phosphatidylethanolamine is a glycerophospholipid that, together with phosphatidylcholine, constitutes more than half of the total phospholipids in eukaryotic cell membranes. We determined that the mutation defined dramatically reduces the enzymatic activity of EPT1, thereby hindering the final step in phosphatidylethanolamine synthesis. Additionally, due to central nervous system inaccessibility we undertook quantification of phosphatidylethanolamine levels and species in patient and control blood samples as an indication of liver phosphatidylethanolamine biosynthesis. Although this revealed alteration to levels of specific phosphatidylethanolamine fatty acyl species in patients, overall phosphatidylethanolamine levels were broadly unaffected indicating that in blood EPT1 inactivity may be compensated for, in part, via alternate biochemical pathways. These studies define the first human disorder arising due to defective CDP-ethanolamine biosynthesis and provide new insight into the role of Kennedy pathway components in human neurological function.

## Teaser

*EPT1* encodes an enzyme involved in the Kennedy pathway of phospholipid biosynthesis, important for cell membrane integrity. Ahmed *et al*. identify an *EPT1* mutation that impairs enzyme activity and causes complex motor neuron degenerative disease. This is the first human disorder shown to arise through Kennedy pathway dysfunction.

## Introduction

**Figure 1:**
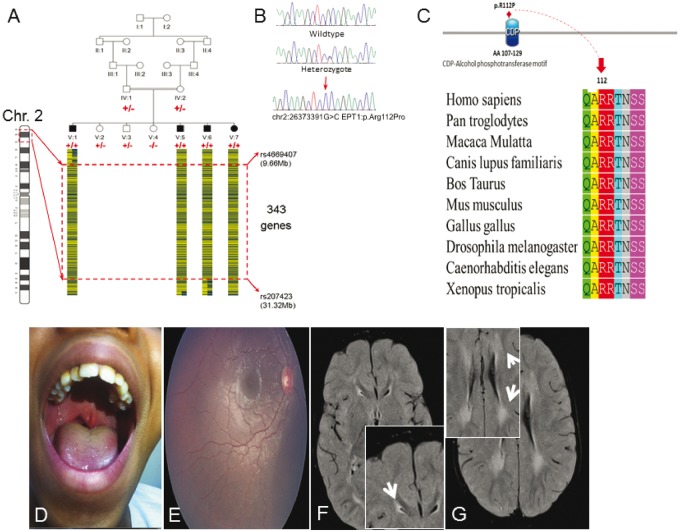
**Genetic and clinical summary of the pedigree investigated.** (**A**) The Omani pedigree with a pictorial representation of the homozygous SNP genotypes across the critical interval (red hashed box) with ‘A’ and ‘B’ genotypes indicated by blue and yellow bars. *SELENOI*/*EPT1* genotypes shown in red (plus sign indicates presence of c.335 G > C alteration, negative sign indicates wild-type). (**B**) Sequence electropherograms showing the DNA encompassing the *SELENOI*/*EPT1* c.335 G > C alteration. (**C**) Species alignment of EPT1 amino acids encompassing the altered p.Arg112 residue showing stringent conservation within the CDP catalytic motif. (**D–G**) Clinical features of affected individuals. (**D**) Photograph of Patient V:1 showing bifid uvula. (**E**) Retinal photograph from the right eye of Patient V:5 showing: mild retinal vessel tortuosity, a dull macular reflex and a mild retinal pigment epithelium level pigmentary disturbance most noticeable inferiorly. (**F** and **G**) Brain MRI scan of Patient V:6 reveals bilateral symmetrical periventricular hyperintensity in the trigon, frontal subcortical white matter and U fibre. All four children displayed similar MRI findings.

Hereditary spastic paraplegia (HSP) encompasses a highly heterogeneous group of disorders characterized clinically by features of upper motor neuron lesion including spasticity, weakness, increased tendon reflexes and upward going plantar reflexes; the term complex HSP is used when these features are associated with other neurological or non-neurological features (Finsterer *et al.*, 2012; Fink, 2013; Lo Giudice *et al.*, 2014; Noreau *et al.*, 2014). Many genes have been implicated in the pathology of HSP, encoding molecules with diverse functional roles (Lo Giudice *et al.*, 2014; Noreau *et al.*, 2014), including a number of genes involved in lipid metabolism (*CYP7B1*, *CYP2U1*, *DDHD1*, *DDHD2*, *BSCL2*, *ERLIN2*, *FA2H* and *PNPLA6*/*NTE*) (Windpassinger *et al.*, 2004; Rainier *et al.*, 2008; Goizet *et al.*, 2009; Alazami *et al.*, 2011; Schuurs-Hoeijmakers *et al.*, 2012; Tesson *et al.*, 2012; Cao *et al.*, 2013; Gonzalez *et al.*, 2013). Here, we investigated four individuals from a single consanguineous Omani family aged between 19 months and 15 years (Fig. 1A) that presented with an unusual neurodegenerative condition best categorized clinically as a complex form of HSP with brain white matter involvement.

## Materials and methods

### Standard protocol approvals, registrations, and patient consents

Informed consent was obtained from participating individuals or their legal guardians and research was performed according to institutional, national and international human subject research guidelines.

### Genetic studies

Genomic DNA samples were extracted from peripheral blood following standard protocols. Genome-wide genotyping was carried out using Illumina HumanCytoSNP-12 v2.1SNP arrays. Data output was visualized in Illumina’s GenomeStudio software. Multipoint linkage analysis was performed assuming a recessive mode of inheritance, full penetrance, and a disease allele frequency of 0.0001 using SimWalk2 (Sobel and Lange, 1996).

Whole exome sequencing was performed by Otogenetics Corp. using the SureSelect Human All Exon V4 (Agilent Technologies) exome enrichment kit on an Illumina HiSeq2000. Reads were analysed on the DNAnexus platform for exome coverage, SNP/InDel variant calling and quality filtering. The exome sequencing produced 21 907 356 mapped 100 bp paired-end reads, matching 95.92% of targeted sequences adequately covered for variant calling (>10× coverage; mean depth, 38.4×).

The identified putative mutations were validated by PCR amplification followed by di-deoxy sequence analysis (Applied Biosystems 3130 DNA Sequencer, Life Technologies). Primer design to amplify the coding exon 5 of the *SELENOI*/*EPT1* gene was done using Primer3 online tool (Rozen and Skaletsky, 2000). Primer sequence specificity was verified by using the UCSC In-Silico PCR tool and their sequences were screened to exclude common single nucleotide polymorphisms.

### Expression of human EPT1 in yeast

Open reading frames encoding full-length wild-type human *SELENOI/EPT1*, and the c.335G > C (p.Arg112Pro) mutated allele identified in patients, were subcloned into the *Saccharomyces cerevisiae* expression vector p416-GPD with a 3’ extension encoding Myc and DDK tags allowing for constitutive expression from the *GPD1* promoter. The TGA codon encoding selenocysteine at amino acid 387 of EPT1 was changed to a cysteine encoding TGT codon by site-directed mutagenesis. Plasmids bearing the wild-type and the mutant allele of human *SELENOI/EPT1* were transformed into the yeast strain HJ091 (*ept1-1 cpt1::LEU2*), which is devoid of endogenous ethanolaminephosphotransferase activity (Henneberry and McMaster, 1999; Henneberry *et al.*, 2000). Yeast cells containing the *SELENOI/EPT1* expression plasmids were grown to mid-log phase and whole cell extracts were prepared and fractionated into soluble and membranous fractions by centrifugation at 100 000 *g* for 1 h. Western blots using anti-DDK antibodies were performed to determine EPT1 protein expression, with Pgk1 (cytosolic fraction) and Dpm1 (membrane fraction) used as loading controls.

### *In vivo* phospholipid radiolabelling

Yeast cells were grown to mid-log phase in 10 ml of synthetic defined medium. Cells were pelleted, washed with synthetic define medium without ammonium sulphate and resuspended in 4 ml of the same medium containing ^14^C-ethanolamine (3 µM, 244 000 dpm/nmol). Cells were cultivated for 1 h at 30°C. At the end of the radiolabelling period cells were harvested, washed with ice-cold water and processed for lipid extraction. Briefly, cells were resuspended in 1 ml of CHCl_3_/CH_3_OH (1:1) and disrupted by bead beating for 1 min at 4°C. The beads were washed with 1 ml of CHCl_3_/CH_3_OH (2:1), and 1.5 ml of H_2_O and 1 ml of CHCl_3_/CH_3_OH (5:1) were added to the combined supernatant to facilitate phase separation. Phospholipids in the organic phase were analysed by thin layer chromatography on Whatman Silica Gel 60A plates using the solvent system CHCl_3_/CH_3_OH/H_2_O/CH_3_COOH (70/30/2/2). Plates were scanned with a BioScan radiolabel imaging scanner, and the bands corresponding to phosphatidylethanolamine (PE) and phosphatidylcholine (PC) were scraped into vials for liquid scintillation counting (Henneberry and McMaster, 1999). Lipid phosphorous was determined as described by Ames and Dubin (1960). Data represent the mean ± standard error of three independent determinations.

### Lipidomic analysis of blood samples

Whole blood from the four affected individuals and their parents (carriers) as well as from five controls was taken for blood phospholipid measurements. Lipid extraction was performed using a modified Bligh and Dyer extraction (Bligh and Dyer, 1959). Briefly, 10 μl of blood was transferred into weighed 2.0 ml Eppendorf tubes. One hundred and eighty microlitres of chilled chloroform/methanol (1:2; v/v) containing internal standards was added. The incubate was vortexed to mix (15 s) with agitation on a thermo mixer (400 rpm) at 4°C in the dark for 1 h (single phase). Sixty microliters of chilled chloroform and 50 μl chilled Milli-Q® water was added and the sample was then vortexed to mix (15 s) and centrifuged at 10 000 rpm for 7 min to separate the phases. The lower organic phase was transferred into clean 2.0 ml microfuge tube (first organic extract). The first organic extract was centrifuged in a vacuum concentrator (SpeedVac) for 10 min. The remaining aqueous phase was re-extracted using 100 μl of chilled chloroform. It was then vortexed to mix (15 s), and centrifuged at 10 000 rpm for 7 min to separate the phases. Lower organic phase was transferred into the first organic extract. The pooled organic extract was dried in a vacuum concentrator (SpeedVac). The lipid extract was resuspended with 200 μl of chilled chloroform/methanol (1:2; v/v). It was then stored at −80°C until mass spectrometry analysis.

For LC/MS analysis, 20 μl aliquots of samples were added into glass vials with glass inserts. A QC sample was generated by pooling 10 μl of each sample together. The sample injection volume was 2 µl. Lipids were separated using gradient elution. Mobile phase A: 40% acetonitrile / 60% 10 mM ammonium formate in H_2_O; mobile phase B: 90% isopropanol / 10% 10 mM ammonium formate in H_2_O. Column: Agilent Zorbax Eclipse plus C18, length: 50 mm, internal diameter: 4.6 mm, particle size: 1.8 µm. Column temperature: 40°C. The flow rate was 0.4 ml/min and the gradient as follows: initial: 20% B, increase to 60% B in 2 min, increase to 100% B in 5 min, stay at 100% B for 2 min, decrease to 20% B in 0.01 mins, keep at 20% B until end of run (10.8 min).

Samples were randomized using Excel for injection into LC/MS instrument and the QC sample was injected after every six samples. To avoid carry over, every QC injection was followed by a blank injection. This injection sequence was repeated three times (technical triplicates). Quantification data were extracted using Agilent MassHunter Quantitative Analysis (QQQ) software. The data were manually curated to ensure that the software integrated the correct peaks. Area under the curve (AUC) of lipids were normalized to AUC of internal standards.

## Results

### Clinical studies

Four children from an extended Omani pedigree aged between 19 months and 15 years and affected by a complex neurodegenerative phenotype. The affected individuals presented in infancy/early childhood with delayed gross motor development, progressive spastic paraperesis and gradual decline in motor function. The oldest affected individual has evidence of upper limb involvement. All affected individuals also manifest an apparently non-progressive mild intellectual impairment. A delay in language acquisition was universal, with dysarthria becoming more noticeable with advancing age. Neuroimaging in all four individuals revealed increased T_2_ intensity signal in the periventricular white matter. The oldest affected child had neurophysiological evidence of a demyelinating peripheral neuropathy; however, upper motor neuron signs predominate over any clinical manifestations of this. Associated variable features included microcephaly, seizure activity and bifid uvula with or without cleft palate. Generalized retinal pigment epithelium level pigmentary disturbance was seen in two of the children, full-field electroretinography, performed in one of these children was consistent with cone-rod dysfunction. Therefore, a clinical diagnosis of complicated hereditary spastic paraplegia had been assigned to these families, although there are additional features present in some individuals that provide evidence of a broader phenotype associated with disturbance of the Kennedy pathway phospholipid cascade (Table 1).

**Table 1:** Clinical features of patients

Patient	V:1	V:5	V:6	V:7
Age at time of assessment (years)	15.06	7.28	3.12	1.60
Sex	Male	Male	Male	Female
Head circumference (SDS/cm)	−3.23 (51.0)	−1.69 (51.0)	−2.03 (48.7)	−3.55 (44.0)
Height (SDS/cm)	−2.24 (149.5)	−2.97 (108)	−2.17 (88.3)	−1.93 (76.2)
Development:				
Gross motor	A few independent steps, gradual decline in motor function	Cruises furniture, gradual decline in motor function	Cruises furniture	Crawling
Speech	Dysarthric, nasal speech, short sentences	Dysarthric, nasal speech, 2– 3-word sentences	A few single words	Babbling
Intellectual disability	Mild	Mild	Mild	
Neurology:				
Upper limb				
Spasticity	✓	x	x	x
Lower limb				
Spasticity	✓	✓	✓	✓
Hyperreflexia	✓	✓	✓	✓
Ankle clonus	✓	✓	x	✓
Extensor plantar responses	✓	✓	✓	✓
Brain MRI	High intensity signal in the periventricular trigonal area with atrophy in surrounding white matter	Increased T_2_ intensity signal in periventricular and subcortical white matter and along optic radiation	Increased T_2_ signal intensity in the periventricular white matter	Increased T_2_ signal intensity in the periventricular region more pronounced around the atria of the lateral ventricles
Nerve Conduction Studies	Motor conduction study of median/ulnar and common peroneal nerves – normal CMAP parameters (amplitude, latency, F-responses and conduction velocity).	Normal (age 5 years)	Borderline prolongation of the median nerve motor latencies, otherwise normal (age 3 years)	Normal
	Median/ulnar and sural nerve sensory study – normal			
	Posterior tibial CMAP amplitude – severely reduced and dispersed, borderline decline in conduction velocity. (age 11 years)			
Ophthalmic phenotype	Reduced visual acuity	Photophobic	Age appropriate visual behaviour	Age appropriate visual behaviour
	No refractive error	Reduced visual acuity	Normal refraction	Mild hyperopic astigmatism
	No further phenotyping	Mild hyperopic astigmatism	Normal anterior segment	Mild retinal vessel tortuosity
		Mild retinal vessel tortuosity	RPE pigmentary disturbance	Dull macular reflex
		Generalized RPE level pigmentary disturbance	No further phenotyping	No further phenotyping
		Dull macular reflex		
		Normal anterior segment		
		ffERG findings of cone-rod dysfunction.		
		No further phenotyping		
Cleft palate/bifid uvula	Bifid uvula, cleft palate	x	High arched palate	Bifid uvula

Height, weight and OFC Z-scores were calculated using a Microsoft Excel add-in to access growth references based on the LMS method (Pan and Cole, 2012) using a reference European population (Cole *et al.*, 1998).

SDS = standard deviation scores; ✓ = presence of a feature in an affected subject; × indicates absence of a feature in an affected subject; CMAP = compound muscle action potential; RPE = retinal pigment epithelium; ffERG = full-field electroretinography.

### Genetic studies

To determine the genomic location of the gene responsible, we undertook genome-wide single nucleotide polymorphism (SNP) genotyping of DNA extracted from blood from family members assuming, autosomal recessive inheritance and that a founder mutation was responsible. This identified a single notable autozygous region of 21.75 Mb on chromosome 2 p, delimited by markers rs4669407 and rs207423 (Fig. 1A and Supplementary Fig. 1), likely to correspond to the disease locus. To identify the causative mutation, we performed whole exome sequencing on an affected family member (Patient V:1). After filtering the identified variants for call quality, potential pathogenicity, population frequency (0.01%) and localization within the candidate interval a single sequence variant located within the disease locus was identified, in ethanolaminephosphotransferase 1 (*SELENOI*/*EPT1*; NM_033505.2; chr2:26,373,391G > C; c.335G > C; p.Arg112Pro; Fig. 1B). The variant affects a stringently conserved arginine residue (p.Arg112Pro; Fig. 1C), which is predicted to be damaging by *in silico* analysis (PolyPhen-2 (Majava *et al.*, 2007) and PROVEAN (Choi *et al.*, 2012), and was found to co-segregate in the family as appropriate for an autosomal recessive condition (Fig. 1A), is not present in online genomic variant databases (1000 Genomes, Exome Variant Server, and Exome Aggregation Consortium (ExAC) and was also absent in 100 regional Omani control subjects.

### Expression of human EPT1 in yeast

**Figure 2:**
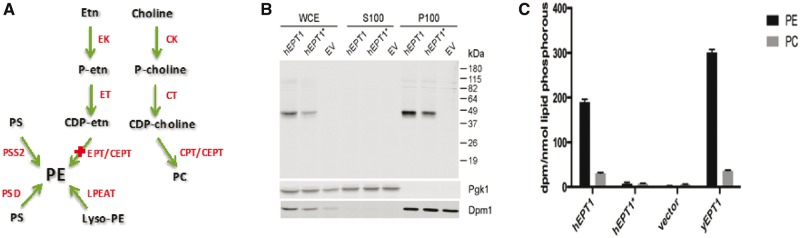
**Outcomes of the p.Arg112Pro EPT1 sequence alteration.** Yeast cells devoid of endogenous ethanolaminephosphotransferase activity were transformed with plasmids bearing wild-type human EPT1 (hEPT1) or EPT1 containing the p.Arg112Pro mutation (hEPT1*) each tagged with a DDK epitope. (**A**) Schematic representation of the CDP-ethanolamine branch of Kennedy pathway showing the role of EPT1 in PE formation. CK = choline kinase; CPT = choline phosphotransferase; CT = phosphocholine cytidylyltransferase; EK = ethanolamine kinase; ET = phosphoethanolamine cytidylyltransferase; Etn = ethanolamine; LPEAT = lyso-PE acyltransferase; PSD = phosphatidyl serine decarboxylase; PSS = phophatidyl serine synthase. (**B**) Western blot versus whole cell extracts (WCE), which were fractionated into soluble (S100) and membrane (P100) fractions, and probed using anti-DDK antibodies. Pgk1 and Dpm1 are soluble and membrane fraction loading and fractionation purification controls, respectively. EV = empty vector control. (**C**) Mid-log phase cells were radiolabelled with ^14^C-ethanolamine for 1 h. As a positive control yeast strain HJ001 (cpt1::LEU2) transformed with an empty vector was also radiolabelled; this strain possess the wild-type genomic allele of yeast EPT1 (yEPT1). Cells were processed for lipid extraction and the radioactivity associated with PE and PC was determined.

To determine the likely pathogenicity of the variant, we next investigated the effect of the p.Arg112Pro mutation on EPT1 activity. *SELENOI*/*EPT1* encodes a CDP-ethanolamine specific enzyme that catalyses the final step in the synthesis of PE via the Kennedy pathway (Fig. 2A) (Horibata and Hirabayashi, 2007). EPT1 belongs to a superfamily of integral membrane phospholipid synthesising enzymes that catalyse displacement of CMP from a CDP-alcohol by a second alcohol with formation of a phosphodiester bond to synthesize a phospholipid. This family of enzymes contains a highly conserved catalytic motif, the CDP-alcohol phosphotransferase motif DG(X_2_)AR(X_8_)G(X_3_)D(X_3_)D (Williams and McMaster, 1998), which for EPT1 is found between amino acid residues 107–129 (^107^DGKQAR^112^RTNSSTPLGELFDHGLD^129^). As the sequence alteration described here affects the highly conserved arginine residue (p.Arg112Pro) within this CDP-alcohol phosphotransferase motif, we examined whether the alteration affects EPT1 catalytic activity. In order to determine this, human EPT1 and mutant EPT1^Arg112Pro^ were expressed from a constitutive promoter in a *S. cerevisiae* strain devoid of endogenous ethanolaminephosphotransferase activity, and their capacity to synthesize PE was determined by metabolic labelling studies. Western blots demonstrated that both human *SELENOI*/*EPT1* alleles were expressed in yeast at comparable levels indicating that protein stability was not affected, were associated with the membrane fraction as would be expected for an integral membrane protein, and exhibited their projected molecular weight of 46 kDa (Fig. 2B).

### *In vivo* phospholipid radiolabelling

We next determined the capacity of human EPT1 and mutant EPT1^Arg112Pro^ to synthesize PE *in vivo.* To do so, the level of radiolabelled ethanolamine incorporated into phospholipid was determined as described previously (Henneberry and McMaster, 1999). Radiolabelled ethanolamine is incorporated into PE by the CDP-ethanolamine pathway, in yeast the PE synthesized by this pathway can be converted to phosphatidylcholine (PC) by PE methyltransferases (Kodaki and Yamashita, 1987; Henry *et al.*, 2012). Thus, the total radiolabel present in the PE plus PC fraction is indicative of the total activity of the ethanolaminephosphotransferase enzyme present. The amount of radiolabelled ethanolamine incorporated into phospholipid for mutant EPT1^Arg112Pro^ was dramatically diminished, being only 3% that of wild-type EPT1 (Fig. 2C). Consistent with this, previous enzyme activity studies of yeast Cpt1 determined that amino acid substitutions at this residue also result in significant decrease in enzyme activity (Williams and McMaster, 1998). Thus, there is a substantive reduction in EPT1 activity due to mutation of p.Arg112 to Pro, consistent with a loss-of-function mutation.

### Lipidomic analysis of blood samples

As we were unable to directly quantify PE levels in brain of affected individuals, and as brain does not efflux PE into the blood, we investigated the amount of PE in blood using mass spectrometry as an indicator for liver EPT1 activity. While there were notable increases in certain individual PE species in patients (e.g. PE36:2, PE36:4 and PE38:5; Supplementary Fig. 2A), there was no significant difference in levels of total PE (Supplementary Fig. 2B), PC, lysophosphatidylcholine (LPC) or phophatidyl serine (PS) (not shown) compared with controls. Given the variation in other individual species between patients, controls and parental carriers, we are not able to determine definitively whether changes to individual biochemical species reflect abnormal biosynthesis as a result of the EPT1 mutation, or coincidental natural variation. Thus while we are unable to assess the outcome of the EPT1 mutation in brain, these findings indicate that in blood a substantive decrease in EPT1 activity does not affect overall PE levels in blood.

## Discussion

Glycerophospholipids are the primary lipid species of eukaryotic cell membranes, of which the major classes include PC and PE (Gibellini and Smith, 2010). PE is normally the second most abundant phospholipid in eukaryotic membranes after PC, constituting 25–45% of phospholipid content (Vance and Tasseva, 2013). PE provides vital structural support to cellular membranes, sustains the function of intrinsic membrane proteins, and is involved in anti-inflammatory, proapoptotic, autophagic, and cell surface attachment functions (Menon *et al.*, 1993; Momchilova and Markovska, 1999; Ichimura *et al.*, 2000; Okamoto *et al.*, 2004; Raetz *et al.*, 2007; Braverman and Moser, 2012; Rockenfeller *et al.*, 2015). PE also plays an integral role in membrane architecture via its unique biophysical properties that are essential for key cell division and membrane fusion processes (Mileykovskaya and Dowhan, 2005; Signorell *et al.*, 2009). These properties are conferred by the shape of PE and its ability to form reverse non-lamellar structures.

The Kennedy pathway is the main biosynthetic route for PE in most mammalian cells including the brain (Zelinski and Choy, 1982; Tijburg *et al.*, 1989; Arthur and Page, 1991). The final step of this Kennedy pathway transfers phosphoethanolamine from CDP-ethanolamine to a lipid anchor such as DAG, and is catalysed by two known enzymes; EPT1 and CEPT1 (Henneberry and McMaster, 1999; Henneberry *et al.*, 2002; Wright and McMaster, 2002; Schuiki and Daum, 2009). Here we identify autosomal recessive p.Arg112Pro alteration of EPT1 in patients with a complex form of HSP. Our enzyme activity studies show a markedly deleterious effect of the substitution on EPT1 catalytic activity. This may be predicted to lead to altered PE fatty acyl Kennedy pathway content and/or synthesis, and the potentially significant alterations to some PE species detected in blood of patients may be consistent with this. Notably, we detected no clear alteration in overall PE content in blood. This may be explained by compensatory activity of CEPT that synthesises PE from the same biochemical source (CDP-ethanolamine), or by the synthesis of PE from PS, either of which may potentially mask changes in specific species PE levels arising due to EPT1 mutation. Thus despite the apparent normalization of total PE levels in blood, potential differences with respect to PE level in the CNS, which could not be assessed in this study, or the abnormalities that we detected in levels of specific PE species in blood, seem likely to account for the clinical features associated with EPT1 mutation. Consistent with this, there is some clinical overlap between the affected individuals described here with those of PE plasmalogen deficiency disorders such as rhizomelic chondrodysplasia punctata (RCDP), a condition characterized by skeletal abnormalities. While there are significant clinical differences between these disorders, cleft palate, spasticity and neuroimaging findings consistent with hypomyelination are features of both conditions, which may be indicative of a common outcome on developmental pathways and neuromorphogenesis due to aberrant PE biosynthesis and reduced levels of plasmalogens, substantial components of myelin.

Taken together, our findings provide new and important insight into the biological role of the Kennedy pathway in mammalian development and neurological function, and document the first human disorder arising due to Kennedy pathway dysfunction.

## Supplementary Material

Supplementary Data

## Abbreviations

EPT1: ethanolaminephosphotransferase 1
HSP: hereditary spastic paraplegia
PC: phosphatidylcholine
PE: phosphatidylethanolamine

## Web resources

BWA (http://bio-bwa.sourceforge.net)

SAMTools (http://samtools.sourceforge.net/)

UCSC Genome Browser (http://genome.ucsc.edu/)

NHLBI Exome Sequencing Project Exome Variant Server (http://evs.gs.washington.edu/EVS/)

Complete Genomics 69 Genomes Data (http://www.completegenomics.com/public-data/69-Genomes/)

PolyPhen-2 (http://genetics.bwh.harvard.edu/pph2/)

PROVEAN (Protein Variation Effect Analyzer) (http://provean.jcvi.org/index.php)

dbSNP (http://www.ncbi.nlm.nih.gov/SNP/)

1000 Genomes Project (http://www.1000genomes.org/)

GATK (http://www.broadinstitute.org/gatk/)

Dindel (https://www.sanger.ac.uk/resources/software/dindel/)

SNP Effect Predictor (http://www.ensembl.org/info/docs/tools/vep/index.html)

Exome Aggregation Consortium (ExAC) Browser (http://exac.broadinstitute.org/)
