# Supplementary material for: A mutation of EPT1 (SELENOI) underlies a new disorder of Kennedy pathway phospholipid biosynthesis
Source: Brain. 2017 Jan 4;140(3):547–54. doi: 10.1093/brain/aww318 (PMC5382949; doi:10.1093/brain/aww318)
Supplement: Supplementary Data [file aww318_supp.pdf]

Supplementary material

Mutation of *EPT1* underlie a new disorder of Kennedy pathway phospholipid biosynthesis

Mustafa Y. Ahmed,[1,*] Aisha Al-Khayat,[2,*] Fathiya Al-Murshedi,[3,*] Amna Al-Futaisi,[4,*] Barry A. Chioza,[1,*] J. Pedro Fernandez-Murray,[5] Jay E. Self,[6] Claire G. Salter,[7] Gaurav V. Harlalka,[1] Lettie E. Rawlins,[1] Sana Al-Zuhaibi,[8] Faisal Al-Azri,[9] Fatma Al-Rashdi,[10] Amaury Cazenave-Gassiot,[11,12] Markus R. Wenk,[11,12] Fatema Al-Salmi,[2] Michael A. Patton,[1,2] David L. Silver,[13] Emma L. Baple,[1] Christopher R. McMaster[5] and Andrew H. Crosby[1]

1 Medical Research (Level 4), University of Exeter Medical School, RILD Wellcome Wolfson Centre, Royal Devon & Exeter NHS Foundation Trust, Barrack Road, Exeter, EX2 5DW, UK

2 Department of Biology, College of Science, Sultan Qaboos University, Sultanate of Oman

3 Department of Genetics, College of Medicine, Sultan Qaboos University, Sultanate of Oman

4 Department of Pediatrics, Sultan Qaboos University Hospital, Sultanate of Oman

5 Department of Pharmacology, Dalhousie University, Halifax, NS, B3H 4H7, Canada

6 Faculty of Medicine, University of Southampton, UK

7 West Midlands Regional Genetics Service, Birmingham Women's NHS Foundation Trust, Mindelsohn Way, Birmingham, B15 2TG, UK

8 Department of Ophthalmology, Sultan Qaboos University Hospital, Sultanate of Oman

9 Department of Radiology and Molecular Imaging, Sultan Qaboos University Hospital, Sultanate of Oman

10 Department of Pediatrics, Sameal Hospital, Ministry of Health, Sultanate of Oman

11 SLING, Life Sciences Institute, National University of Singapore, Singapore

12 Department of Biochemistry, National University of Singapore, Singapore

13 Signature Research Program in Cardiovascular and Metabolic Disorders, Duke–NUS Medical School, Singapore

*These authors contributed equally to this work.

Correspondence to: Professor Andrew H. Crosby,
Medical Research (Level 4),
RILD Wellcome Wolfson Centre,
Royal Devon & Exeter NHS Foundation Trust,
Barrack Road, Exeter, EX2 5DW, UK.
E-mail: A.H.Crosby@exeter.ac.uk

Correspondence to: Dr Emma L. Baple,
Medical Research (Level 4),
RILD Wellcome Wolfson Centre,
Royal Devon & Exeter NHS Foundation Trust,
Barrack Road, Exeter, EX2 5DW, UK.
E-mail: E.Baple@exeter.ac.uk

Supplementary figure 1

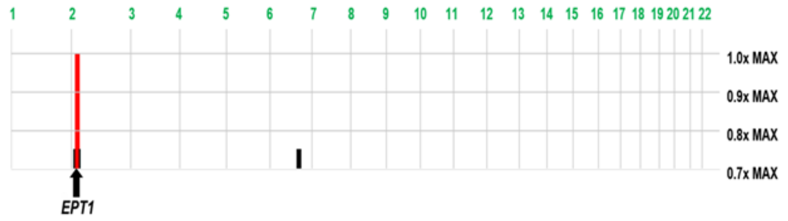

Supplementary figure 1

Homozygosity map of affected family members. Homozygosity plots indicate detected autozygous genomic regions shared between affected family members with the homozygous region containing *EPT1* indicated in red.

Supplementary figure 2

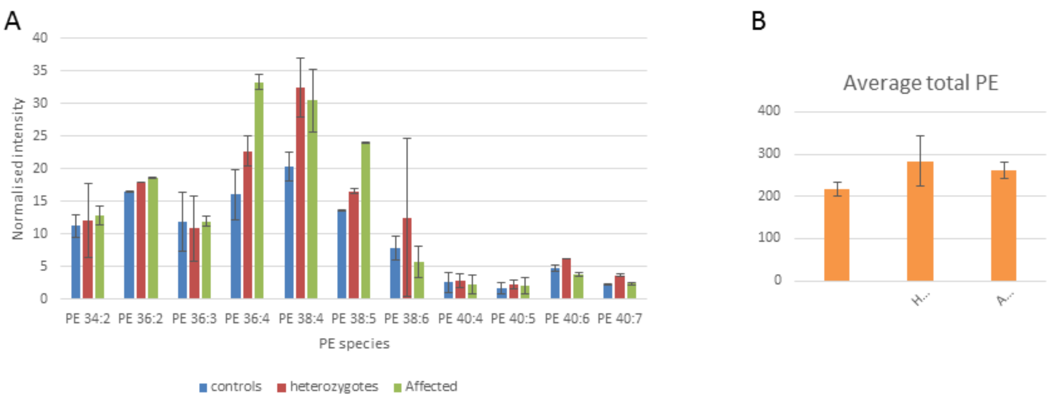

Supplementary figure 2

A) Normalised intensity of all blood PE species measured by LC-MS/MS in affected vs control (wild type or parental heterozygote) individuals. **B**) Total levels of PE in affected individuals versus control (wild type or parental carrier) individuals.
